# Light and Heat as Causes of Cataract

**Published:** 1903-02-07

**Authors:** 


### LIGHT AND HEAT AS CAUSES OF CATARACT.

A most interesting observation has been made by Dr. William Robinson,[1] of Sunderland, in regard to the frequency with which hard cataract is found among bottle-finishers, men whose work requires them to expose their eyes very frequently to the intense light and heat of molten glass. How much commoner cataract is among these men than among the general population may be gathered from the fact that 18 out of 75 hard cataracts operated on last year in the Sunderland and Durham Eye Infirmary were in bottle-finishers, of whom there are only about 200 to 300 in a population of nearly a million and a quarter in the county.

"The bottle-finisher, holding the bottle on the 'punty' in his left hand, with an iron rod in his right, looks directly with both eyes into the sea of molten glass, and takes just sufficient to form the rim on the neck of the bottle. . . . The extent to which the eyes of the bottle-finishers are exposed to the most brilliant light and the intensest heat from the furnace may be estimated by the fact that he generally works five shifts each week, a shift lasting 11 hours, and at each shift he finishes 110 to 120 dozen bottles at the rate of two and a half bottles per minute. The time in which he is obliged to look into the furnace while he gets the metal for the rim is about three seconds, so that at each shift his eyes are exposed to the glare of the furnace for at least 66 minutes, or about five and a half hours a week."

Both eyes are practically always affected. The disease begins comparatively early in life—as often as not before the age of 50, and not uncommonly before 40 even—and progresses slowly. The opacity first appears at the posterior pole of the lens, immediately under the posterior capsule, and is shaped like a disc or coin, which by oblique illumination is distinctly brass-coloured, and as the haziness spreads it at first clings closely to the posterior capsule so that it is saucer shaped during the earlier stages of the disease. "I do not deny," says Dr. Robinson, "that an ordinary senile cataract may begin as a posterior polar cortical cataract; if it does so it is a rare event, whereas practically all cases in bottle-finishers begin in this way." The disease is undoubtedly due, he says, to to the great light and heat of the furnace, and the reason why the mischief always begins at the posterior pole of the lens is that the nodal point (which practically corresponds to the optical centre) is situated at the exact spot where the cataract begins. Here all the principal rays of the various pencils of rays falling on the lens cross and pass without refraction, so that at this point the lens receives the brunt of all the direct rays, harmful by their intensity and heat from the furnace, and is therefore the first point to suffer injury. Moreover, other rays which have been refracted at the anterior surface of the lens become crowded on to the same area on the posterior surface, and still further the bright light of the furnace contracts the pupil so that the iris shields the periphery of the lens at first from damage. Dr. Robinson feels confident that the disease might be prevented by the workmen wearing dark blue spectacles, but when advised to do so the bottle-finishers offer various excuses. There can, however, be no doubt that all men whose work compels them to expose their eyes to such strong light and heat ought to wear glasses, as, for example, is done in steelworks and by men employed in certain electro-metallurgical processes.

[1] Brit. Med. Jour., Jan. 24.

